# Supplementary material for: Upstream modes and antidots poison graphene quantum Hall effect
Source: Nat Commun. 2021 Jul 12;12:4265. doi: 10.1038/s41467-021-24481-2 (PMC8275581; doi:10.1038/s41467-021-24481-2)
Supplement: Supplementary file 1 — Supplementary Information [file 41467_2021_24481_MOESM1_ESM.pdf]

Supplementary information for Upstream modes and antidots poison graphene quantum Hall effect

N. Moreau[1], B. Brun[1], S. Somanchi[2], K. Watanabe[3], T. Taniguchi[3], C. Stampfer[2] & B. Hackens[1]

[1]IMCN/NAPS, Université catholique de Louvain (UCLouvain), B-1348 Louvain-la-Neuve, Belgium

[2] JARA-FIT and 2nd Institute of Physics - RWTH Aachen, Germany

[3] National Institute for Materials Science, Namiki, Japan

Supplementary Note 1: Fan diagrams and electon-hole assymetry

In this work, we studied two samples (G1 and G2). Both consist in hBN-encapsulated monolayer graphene etched in a constriction-shaped geometry as shown with optical microscopy photographies in the inset of Supplementary Fig. 1a for sample G1 and in Supplementary Fig. 1b for sample G2. The charge carriers density n in graphene is tuned via the back gate voltage V_{bg} applied on the degenerately doped silicon substrate separated from graphene by a 300 nm-thick layer of silicon oxide and the 20 nm-thick bottom hBN. The lever arm $\alpha = n/V_{bg}$ can be extracted for both samples by recording longitudinal resistance R_{xx} while varying V_{bg} (the Fermi energy E_F is shifted) and the magnetic field B (the energy gap between the Landau levels (LLs) depends on B). In a textbook representation of quantum Hall effect, backscattering is maximized when a E_F is aligned with one of the LL. It results in strips of local R_{xx} maxima in the $V_{bg} - B$ map located along the straight lines

$$B_n = \frac{\pi\hbar}{2en}\alpha(V_{bg} - V_{bg,0}) \tag{1}$$

where n are the LLs labels (negative for holes and positive for electrons) and $V_{bg,0}$ is the back gate voltage corresponding to charge neutrality (+1.2 V for sample G1 and +7.4 V for sample G2). Fits to the R_{xx} maxima strips were performed for both samples (dotted lines in Supplementary Fig. 1a for sample G1 and Supplementary Fig. 1c for sample G2) and allowed to determine the values of α about 9.4×10^{14} m^{-2}V^{-1}.

In the manuscript, such as in Supplementary Note 2, all the presented results were obtained with hole type charge carriers. In this section, we explain why we were not able to lead a proper study with electron type charge carriers at high magnetic field in both studied samples.

In sample G1, it was not possible to properly define the longitudinal resistance R_{xx} at high magnetic field for electron-type charge carriers, as shown in Supplementary Fig. 1a. Indeed, the current dropped to zero between $\nu = 0$ and $\nu = +4$, leading to diverging R_{xx}. It must be due to the loss of electrical contact between the metallic leads and the graphene plane. For $\nu > +4$, we recovered all the current we injected in the sample but the voltage V_{xx} exhibited large chaotic fluctuations that we associate to poor equilibration of the metallic contacts with the QH QHECs. The SGM maps obtained with these parameters were therefore unexploitable. Nevertheless, we

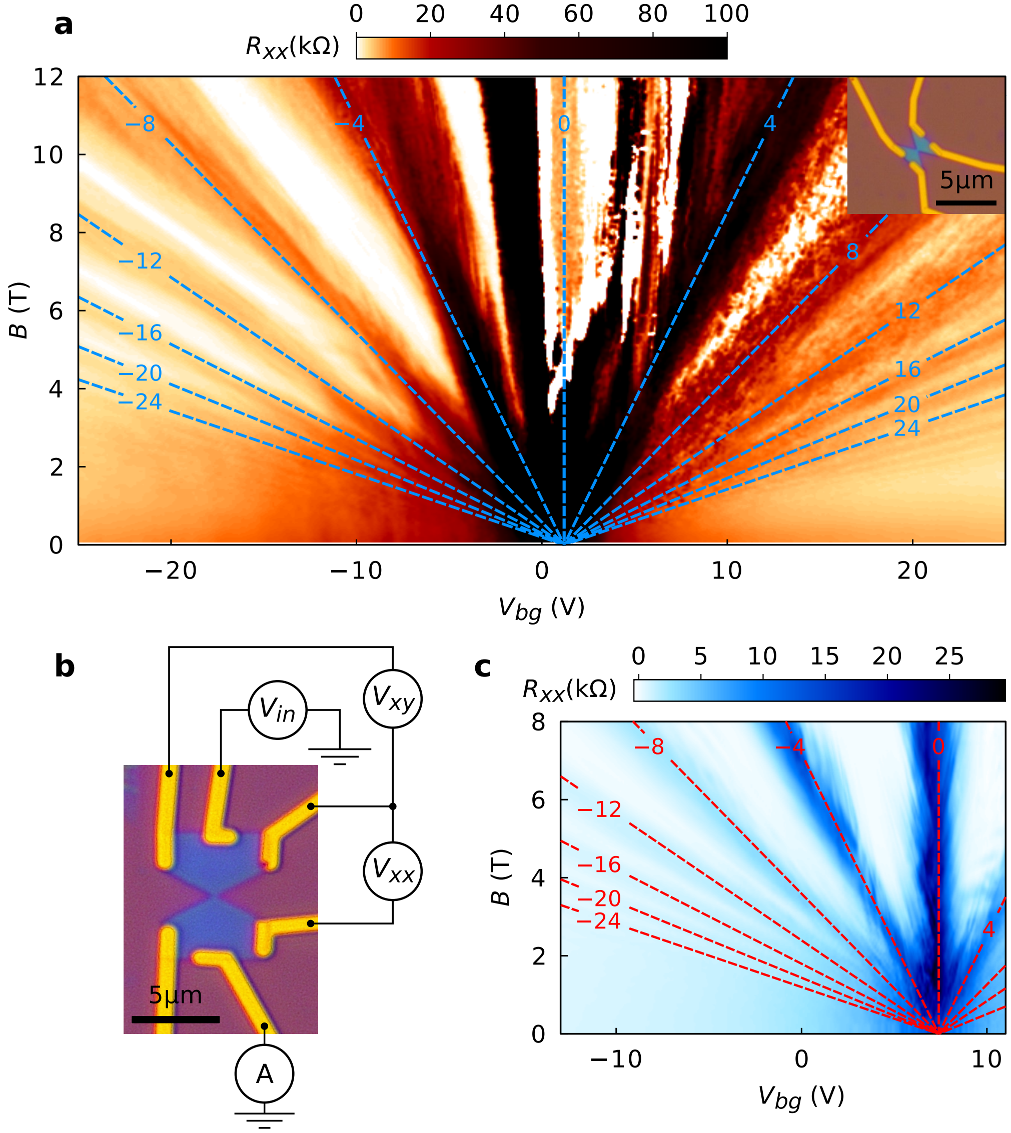

Supplementary Figure 1: Fan diagrams for samples G1 and G2 a, R_{xx} map as a function of V_{bg} and B for sample G1. The dashed lines follow the couples (V_{bg}, B) for which, in a textbook representation of quantum Hall effect, a bulk Landau level is aligned with the Fermi energy (R_{xx} exhibits local maxima along these lines). These maxima coincide with the filling factors ν that label each line. The inset shows an optical picture of sample G1. The BN-encapsulated graphene device has a blue color and the 4 gold contacts have a yellow color. **b**, Optical picture of sample G2. The 6 contacts of this sample allow to measure both longitudinal resistance R_{xx} and Hall conductance G_{xy}. **c**, R_{xx} map as a function of V_{bg} and B for sample G2. As in **a**, the dashed lines are labeled by the filling factor.

managed to extract data in the electron side at lower magnetic field that are discussed in [1]. In this paper, we discuss the role of contacts in the electron-hole asymmetry.

In sample G2, a leak between the electron gas and the back gate for $V_{bg} > +11$ V prevented us to make measurements with electron-type charge carriers.

Supplementary Note 2: SGM measurements and tip potential screening

In this section, we first present the SGM measurements obtained with sample G2. Peculiar SGM signatures appear in this device at low tip voltage, that we attribute to screening effects of the tip potential by the back gate. Then, we detail the resonance condition enabling the coupling between counterpropagating quantum Hall edge channels (QHECs) through an antidot. Finally, we further discuss the interplay between the tip, the back gate and the graphene plane and their mutual screening. It has important implications in the characterization of the tip-induced potential.

2.1 SGM measurement on sample G2

All experimental data presented in the manuscript were obtained on sample G1. In this section, we present the SGM measurements obtained on our second device (sample G2). The data exhibit the same features as presented in the manuscript. A schematic of the experimental setup used for sample G2 is shown in Supplementary Fig. 2a. The longitudinal resistance R_{xx} as well as the Hall conductance G_{xy} recorded as a function of back gate voltage V_{bg} under a magnetic field of 14 T are presented in the graphics of Supplementary Fig. 2b.

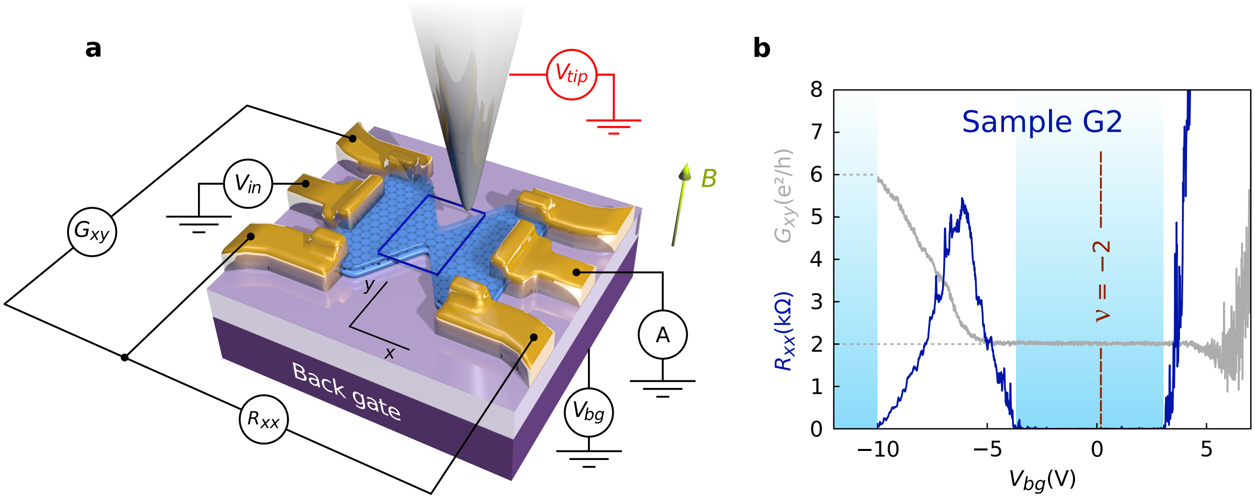

Supplementary Figure 2: Experimental setup for sample G2 a, Schematic of sample G2. The biased tip can locally change the electron density when applying the voltage V_{tip} and is moved at a distance $d_{tip} \sim 50$ nm above the encapsulated graphene constriction. The global electron density is tuned by the back gate voltage V_{bg}. A perpendicular magnetic field B is applied perpendicularly to the graphene plane. **b**, Transport measurements (the longitudinal resistance R_{xx} in blue and the Hall conductance G_{xy} in gray) obtained for $B = 14$ T. The light blue stripes indicate the region of zero R_{xx}, associated with conductance plateau at $2e^2/h$ around $\nu = -2$ and $6e^2/h$ around $\nu = -6$.

Figure 3 presents several SGM maps obtained at 14 T by scanning the tip at ~ 55 nm above the graphene plane within the scan area sketched in Supplementary Fig. 2a. As for the manuscript's data, we explored the transition between $\nu = -6$ and $\nu = -2$ (Supplementary Fig. 3a) where breakdown of QHECs topological protection occurs. In these conditions, the same fingerprints of high resistance rings along the device edges emerge in sample G2, highlighting the presence of antidots as the cause of topological breakdown. In the manuscript (sample G1), we explain

that the rings are centered above the antidots, at a distance between 50 and 150 nm from device borders, in agreement with the QHECs region width reported in literature [2]. In Supplementary Fig. 3b, however, the rings are centered on the device's borders. In figures 3c-f, SGM signatures are even not circular and exhibit peer shapes.

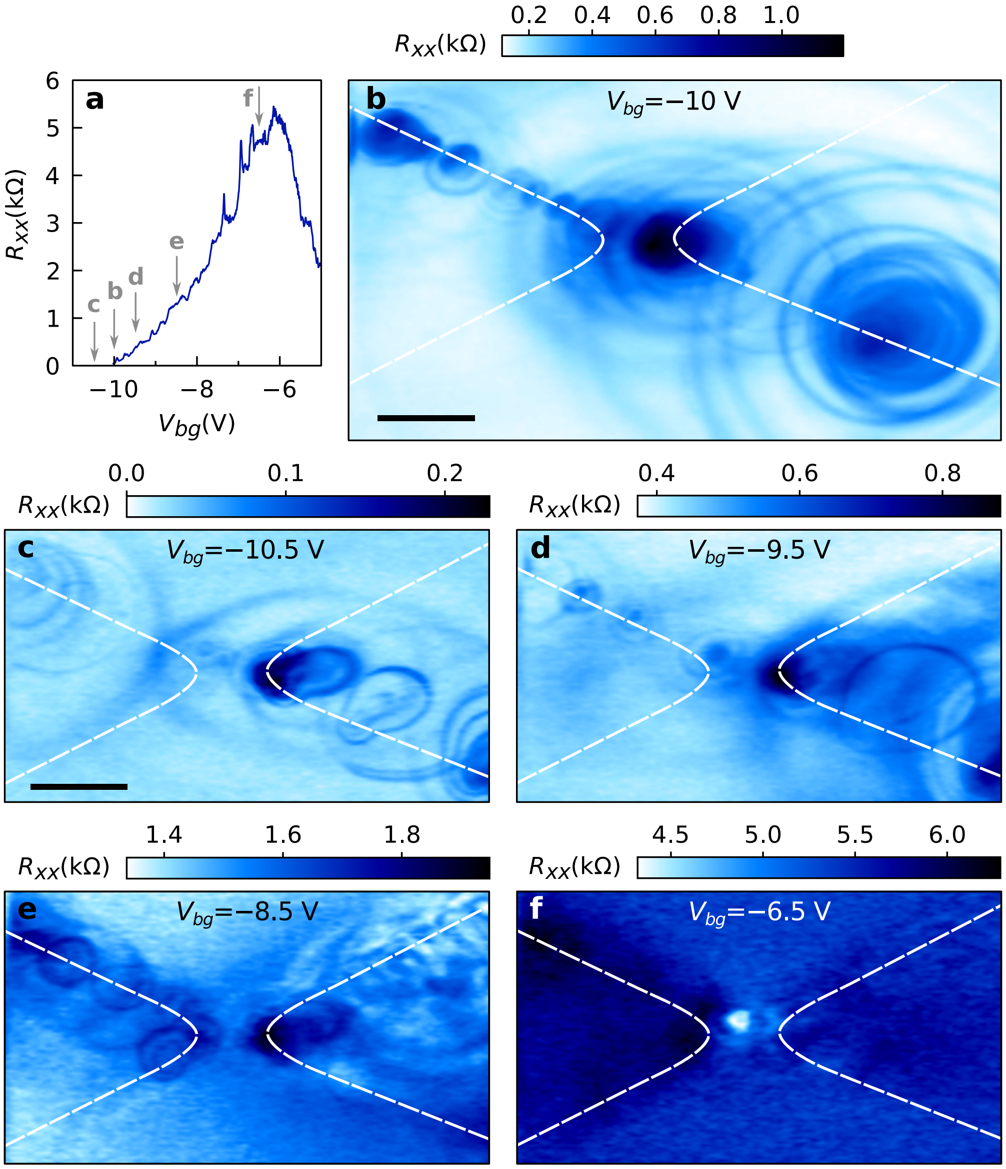

Supplementary Figure 3: Imaging the break-down of topological protection in sample G2. **a**, Zoom on Supplementary Fig. 2 between $\nu = -6$ and $\nu = -2$ corresponding to the loss of topological protection (non-zero R_{xx}). **b-f**, SGM maps obtained at 14 T and $V_{tip} = +3$ V (**b**) and $V_{tip} = 0$ V (**c-f**). The V_{bg} values used for each map are indicated with the arrows in **a**. The rings of higher R_{xx} are the loci of tip positions leading to a resonance between an antidot localized state and the counterpropagating QHECs and hence the beakdown of topological protection of these channels.

We explain these surprising observations by two mechanisms:

- Metallic gates and graphene are known to screen the tip potential, leading to shifts and deformations of the SGM signatures. In particular, Schnez et al. studied a graphene quantum dot with SGM and observed that the circular signatures in SGM maps, caused by Coulomb blockade, were shifted because of the side gates used to control the charging state of the dot [3]. We interpret our observed shifts and deformation of high R_{xx} rings as screening of the tip-induced potential by the back gate. We are comforted in our interpretation by the fact that deformations of the high R_{xx} rings are stronger at low V_{tip} (Supplementary Figs. 3c-f with $V_{tip} = 0$ V) than at high V_{tip} (Supplementary Fig. 3b with $V_{tip} = +3$ V) where rings are circular. Screening effects will be discussed in more details in Supplementary Note 2.3.

- There is an uncertainty on the position of sample G2 edges coming from the fact that they were determined by electrostatic force microscopy. This technique consists in mapping the force applied on the tip, related to the electrostatic interactions with the substrate. When the tip lies above graphene or above the remote polarized back gate, both grounded metallic plane but located at different distances from the tip, the resulting electrostatic interaction in different. The transition of electrostatic environment when crossing the edge result in a smooth signal when the tip is 55 nm above the graphene plane and hence an uncertainty of about 100 nm in our case. In sample G1, edges positions were determined more conventionally by atomic force microscopy (AFM) and the sharp transition of the measured height at the edges decreases the uncertainty on their position.

2.2 Resonance condition

Here, we detail the resonance condition of an antidot, whose electronic structure has been extensively studied in two recent papers [4, 5] (note that in these cases antidots were created by introducing charges in the hBN substrate whereas they preexist in our samples). When the antidot charging energy is equal for N and $N+1$ charge carriers, charge-discharge events can occur. In the QH regime, the antidot charging energy is given by [6–9]

$$U(N) = \frac{1}{2C_\Sigma}\left(qN - qf_c\frac{B\delta A}{\phi_0} + C_{tip}V_{tip} + C_{bg}V_{bg}\right)^2 + \sum_{n=1}^{N} E_n \tag{2}$$

where C_Σ is the antidot capacitance, q the electron ($-e$) or hole ($+e$) charge, f_c the number of QHECs around the antidot, δA the area variation of the antidot, $C_{tip}V_{tip}$ and $C_{bg}V_{bg}$ the charge imbalance induced by the tip and the back-gate voltage respectively and E_n the quantum resonant energy levels. By moving the biased tip near the antidot, the capacitance term C_{tip}, the area δA as well as the quantum terms E_n in Supplementary Eq. (2) vary according to the tip position $\mathbf{r}_{tip}$. The Coulomb rings in Figs. 1c-d of the manuscript and Supplementary Figs. 3b-f are the loci of $\mathbf{r}_{tip}$ for which the resonance condition $U(N) = U(N+1)$ is reached, when the system is at equilibrium. As will be discussed in Supplementary Note 7, the quantum terms are small compared to the Coulomb contribution.

2.3 Characterization of the tip-induced perturbation

In Fig. 3b of the manuscript, we showed that mapping R_{xx} vs V_{bg} and y_{tip} (along a line passing above an antidot) yields a direct image of the tip-induced potential felt by the antidot for a given

V_{tip}[1]. Indeed, the induced potential was given by the shift in the Coulomb resonance signatures (R_{xx} peaks) along V_{bg} corresponding to the alignment of one of the antidot resonance levels with the Fermi energy. We then used Lorentzian functions to fit the induced potential and the function turns out to decay faster when the tip is above the graphene plane, which is expected from the screening of the graphene charge carriers. Here, we explore in more details the influence of screening on the tip-induced potential.

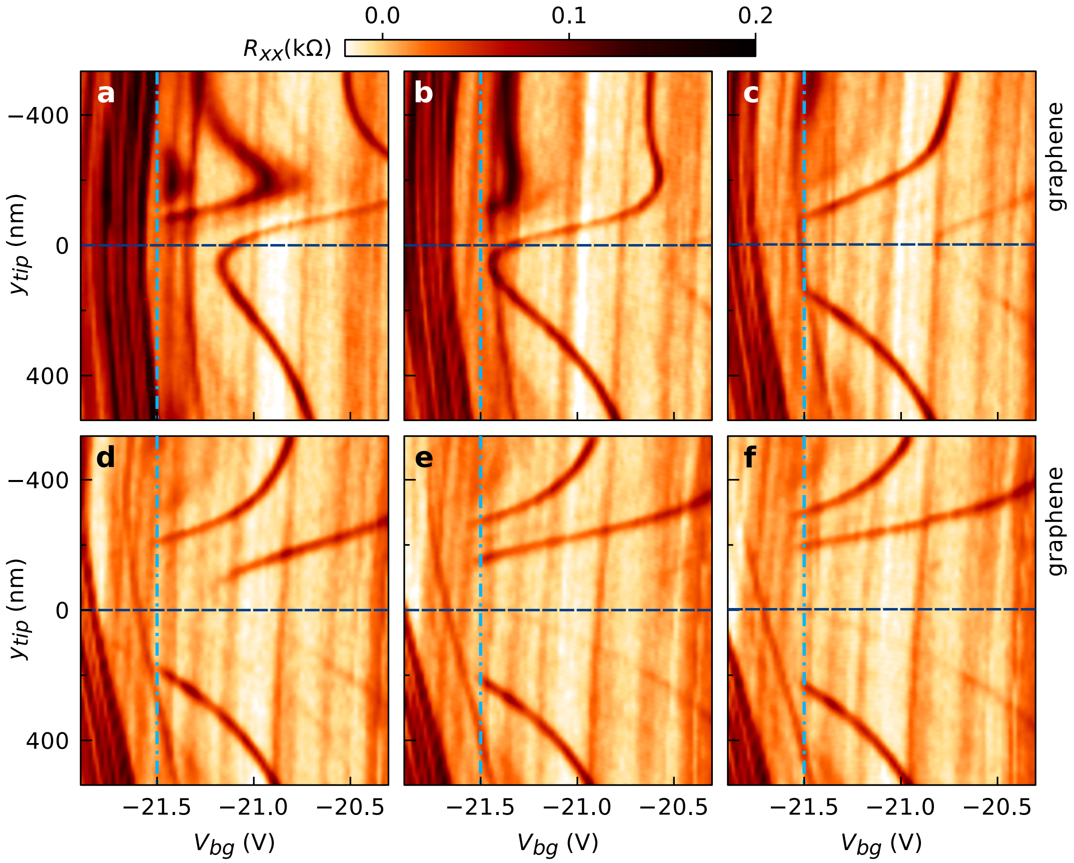

Supplementary Figure 4: Effect of the screening on the tip-induced potential a-f, R_{xx} maps when varying y_{tip} and V_{bg} obtained in the same conditions as Fig. 3 of the manuscript with $V_{tip} = -3$ V (**a**), -2 V (**b**), -1 V (**c**), 0 V (**d**), $+1$ V **e** and $+2$ V (**f**). Coulomb resonance signatures corresponding to the studied antidot all disappear when $V_{bg} < -21.5$ V (light blue dash-dotted lines), as discussed in the manuscript. The dark blue dashed lines indicate the device edge position.

Figure 4 gives the same maps as Fig. 3b of the manuscript for different values of V_{tip}. For each map, we expect Coulomb resonances to follow a Lorentzian evolution along y_{tip} with an amplitude related to V_{tip}, as made in the manuscript. A striking feature however appears in Supplementary Figs. 4a,b. When following a Coulomb resonance line, it first reaches a maximum then a minimum value and the curvatures changes between them. In other words, the tip-induced potential exhibits a positive biased feature when the tip is outside the graphene plane and a negative biased feature when the tip is above graphene, far from the expected Lorentzian evolution. Schnez

[1]The workfunction difference between the tip and graphene plane should be added to the tip voltage so that around +3 V should be added to the effective V_{tip} used in Supplementary Eq. (2) [10]

et al. observed the same kind of signature and attributed it to two different origins. First, the coupling between the tip and the studied antidot (C_{tip} in Supplementary Eq. (2)) is not symmetric on both sides of the antidot because of the difference of screening between the back gate and the graphene plane. Second, the coupling between the back gate and the antidot (C_{bg} in Supplementary Eq. (2)) also changes with the tip position due to screening effects. The combination of both effects explain how the features of Supplementary Figs. 4a,b emerge.

As discussed in the manuscript, the resonance lines vanish below a given V_{bg}. In Supplementary Fig. 4, the V_{bg} limit is the same in all maps and is indicated with blue dashed lines.

Finally, some resonances are also visible for $V_{bg} < -21.5$ V in Supplementary Figs. 4 and in Fig. 3 of the manuscript. These resonances evolve slightly with the tip position. We ascribe them to signatures of the topological breakdown occurring between the $\nu = -6$ and $\nu = -10$ plateaus, far from the region scanned by the tip in Fig. 1 of the manuscript. It explains the small influence of the tip on these signatures.

Supplementary Note 3: Coulomb diamonds

In this section, we show that the high R_{xx} lines observed in our SGM data are indeed caused by transport through localized states (here associated with the presence of antidots). We drive this conclusion from Supplementary Fig. 5c obtained by varying a DC bias voltage V_{sd} (resulting in the measured DC current I_{sd} through the sample) while moving the tip along the red dashed line in Supplementary Figs. 5a,b. When applying a DC bias, the electrochemical potential levels of the input (source) and output (drain) leads are shifted with respect to each other and the energy window in which a discrete energy level associated with an antidot can contribute to charge carrier conduction is widened. It yields in the appearance of diamond shaped bright areas in the R_{xx} map of Supplementary Fig. 5c, indicating the couples of V_{sd} and tip position for which transport through the antidot is forbidden (Coulomb blockade) so that counterpropagating QHECs are not coupled (no backscattering). These diamonds are separated by non-zero R_{xx} regions corresponding to resonance signatures (highlighted with blue arrows). In that situation, charge carriers can flow through the antidot and counterpropagating QHECs are coupled.

The same kind of low-R_{xx}-diamonds is obtained when varying both I_{sd} and V_{bg} as shown in Supplementary Fig. 6b. Contrarily to Supplementary Fig. 5c where the diamonds are associated to a single antidot[2], tuning V_{bg} changes the charging energy of all the antidots in the system. The non-zero R_{xx} regions between each diamond correspond to the R_{xx} peaks observed at zero DC bias in the inset of Supplementary Fig. 6a. It is therefore clear that these peaks are signatures of localized states resonances associated with antidots.

Supplementary Note 4: Emergence of counterpropagating QHECs

In the manuscript, we show that the topological breakdown of QHECs is caused by the presence of both forward and backward QHECs along the same graphene edge and their coupling through antidots. In this section, we discuss the most widespread hypotheses about the appearance of

[2]the resonance conditions are reached by changing the charging energy of this antidot only when moving the tip in its vicinity

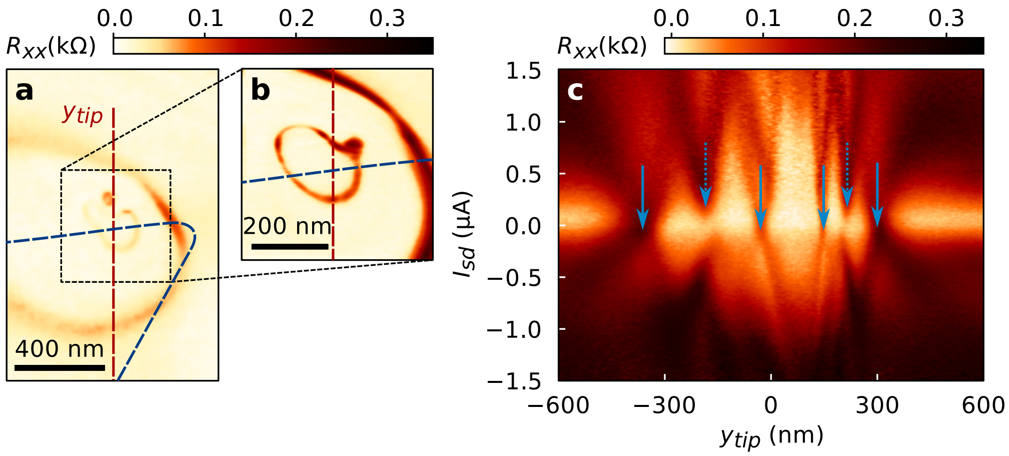

Supplementary Figure 5: Coulomb diamonds related to a single antidot a, SGM map obtained at 12 T with $V_{bg} = -17.75$ V and $V_{tip} = 0$ V in sample G1. The blue dashed line indicate sample edges. **b**, Zoom in the square area indicated with a dotted line in **a**. Coulomb rings are more contrasted due to slower scan rate. **c**, R_{xx} map as a function of the tip position (along the red dashed line y_{tip} in **a**) and the source-drain DC current I_{sd}. Blue arrow indicate the resonance signatures (Coulomb rings in **a** and **b**). These resonances widen by increasing the absolute value of I_{sd} so that diamond-shaped regions of zero R_{xx} appear in the graph. The dotted arrows indicate weaker resonances not visible in **a** and **b**.

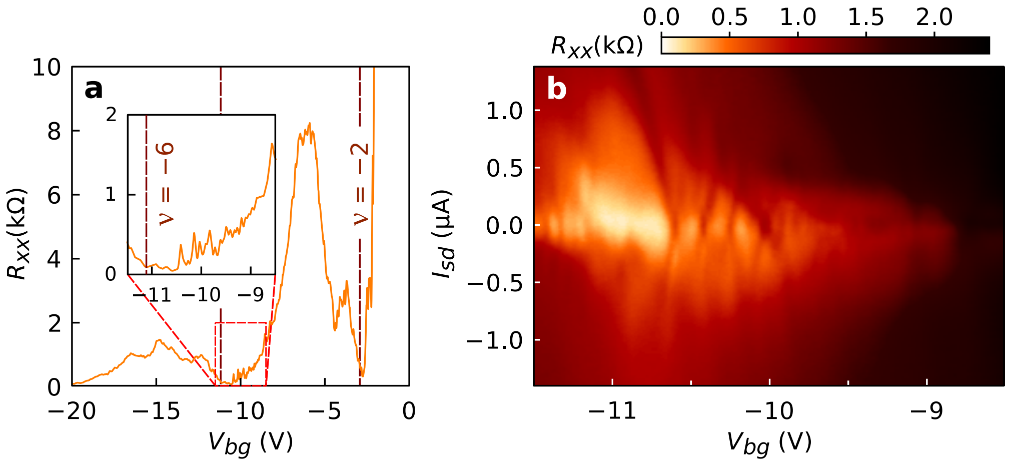

Supplementary Figure 6: Coulomb diamonds obtained when varying back gate voltage a, R_{xx} measured as a function of V_{bg} at 8 T in sample G1. The inset shows a zoom around $\nu = -6$ (red dashed line). R_{xx} signal deviates from zero (loss of the QHECs topological protection) by exhibiting peaks. **b**, R_{xx} map as a function of V_{bg} and I_{sd}. Zero R_{xx} Coulomb diamonds appear between each R_{xx} peak of **a**.

these counterpropagating channels. We point out the different unsolved issues in these theories and the additional explorations that they required.

In 2008, Silvestrov and Efetov [11] reported in a theoretical article that the screening of the back gate (modeled by an infinite conductor plane) by graphene charge carriers lead to the emergence of charge accumulation along the device edges. In the QH regime, it results in the appearance of counterpropagating QHECs along device borders. This theory has been widely invoked by several authors to explain experimental observations in the QH regime [2,12,13].

In 2013, Vera-Marun et al. studied the evolution of the lever arm as a function of the distance from edges in graphene (and bilayer graphene) samples [12]. To do so, they applied a perpendicular magnetic field that confined charge carriers along the device edges, in a region whose width is given by the cyclotron radius. Hence, they were able to probe the lever arm evolution between the bulk and the edges by changing the cyclotron radius of the charge carriers participating to conduction. Their results were close to theoretical simulations based exclusively on electrostatics, excepted when the cyclotron radius becomes smaller than 25 nm where the lever arm seamed to saturate. The lever arm increased by $\sim 100\%$ between zero and high magnetic field.

In 2014, Barraud et al. conducted a similar study but found an increase of the lever arm of only 20% between zero and maximal magnetic field [13]. They concluded that the charge density profile is not given solely by electrostatics. They claim that features depending on the studied sample, such as localized states at the edges, can reduce the theoretical charge accumulation due to electrostatics.

In 2016, Cui et al. observed that Hall conductance plateaus (corresponding to zero R_{xx}) don't necessarily coincide with an insulating bulk in graphene samples [2] as expected in conventional QH effect observed in semiconductor-based two dimensional electron gas [14]. To explain this surprising discovery, they use the charge accumulation model and the associated presence of counterpropagating channels in the QH regime. They state that QHECs region (where live forward and backward channels coexist in close proximity) is decoupled from the bulk by an incompressible (insulating) strip. Backscattering can therefore occur between counterpropagating channels along the same edge while the bulk is insulating or backscattering can be prohibited while the bulk is conducting. However, the shift they observed between Hall plateau and insulating bulk positions as a function of V_{bg} is not always the same for holes and electrons. Their conclusion was the same as Ref. [13] : impurities at the edges locally modulate the electrostatic potential at the edges.

Finally, in 2019, Marguerite et al. [15] used a SQUID-on-tip nanothermometer (that can also be used as a SGM) to study the breakdown of QHE topological protection in graphene. As in the present study, they observed signatures of large longitudinal resistance at the edges and invoked the presence of counterpropagating QHECs to explain their results. They however made a striking observation : at the charge neutrality point ($V_{bg} = 0$), signatures of counterpropagating QHECs persist. Inhomogeneous gating is not able to explain this observation since the increase of charge carriers density at the edges should be proportional to the applied V_{bg} (here close to zero). They then conclude that a holes accumulation at graphene edges preexist in their samples, due to the presence of negatively charged impurities at the edges.

From these different studies, it appears that the exact shape as well as the physical origin of the inhomogeneous edge potential remain largely unknown. For our devices, the lever arm modulation is small when varying the magnetic field (the stripes of local resistance maxima only slightly deviate from the theoretical dashed straight lines in Supplementary Figs. 1a,c). This could be explained either by the presence of edge impurities affecting the potential modulation (Barraud et al. and Cui et al.) so that it saturates quickly near the physical device's borders or by the presence of a preexisting hole accumulation (Marguerite et al.). These two hypothetic mechanisms can furthermore coexist.

In conclusion, further studies are needed to characterize accurately the influence that edge impurities have on the potential modulation. In this work, we therefore use an arbitrary potential for simulation that exhibits qualitative features compatible with experimental evidences. That is, a bending of edge potential that quickly saturates when approaching device's borders (further

details in Supplementary Note 7).

Supplementary Note 5: Finding the maximal tip-induced density change

In Fig. 4b of the manuscript, we converted the tip bias V_{tip} into an induced change of carrier density. In particular, since we deal with hole carriers and we apply a positive tip bias, we induce a maximum decrease of the local hole density denoted $|\Delta n_{tip}|$. To convert V_{tip} into $|\Delta n_{tip}|$, we can rely on the maps of Supplementary Fig. 4. The shift of the Coulomb resonances gives a direct correspondence between the effective tip-induced potential φ_{tip} and V_{bg} as a function of y_{tip}. Because the result of Fig. 4b in the manuscript were obtained with the tip above the antidot, $|\Delta n_{tip}|$ corresponds to the maximal change of density, obtained at the maximum of φ_{tip}. For each value of V_{tip} of Supplementary Fig. 4, we then have

$$\varphi_{tip}^{max}(V_{tip}) = \Delta V_{bg}(V_{tip}) \tag{3}$$

where $\Delta V_{bg}(V_{tip})$ is the difference between the maximal Coulomb resonance shift in V_{bg} and the Coulomb resonance position in V_{bg} without tip influence ($|y_{tip}| = \infty$). In Supplementary Eq. (3), the term $\varphi_{tip}^{max}(V_{tip})$ is supposed to be proportional to V_{tip} [16]. However, we must notice that $\Delta V_{bg}(V_{tip} = 0) \neq 0$ in Supplementary Fig. 4c (and Fig. 3b of the manuscript). This is due to the workfunction V_{WF} between the tip and the graphene plane that adds an offset to the applied tip bias V_{tip} so that

$$\Delta V_{bg}(V_{WF}) = 0 \tag{4}$$

The maximal tip-induced potential is then given by $\varphi_{tip}^{max}(V_{tip}) = \alpha_{tip}(V_{tip} - V_{WF})$ with

$$\alpha_{tip} = \frac{\Delta V_{bg}(V_{tip})}{V_{tip} - V_{WF}} \tag{5}$$

By using the result of Supplementary Eq. (1), $|\Delta n_{tip}|$ can be obtained from Supplementary Eq. (3) as

$$|\Delta n_{tip}| = \alpha |\Delta V_{bg}(V_{tip})| \tag{6}$$

Based on Supplementary Eq. (6), it is easy to link $|\Delta n_{tip}|$ to V_{tip}. From Eqs. 6 and 5, we have

$$|\Delta n_{tip}| = \left| \frac{\alpha}{\alpha_{tip}} \right| (V_{tip} - V_{WF}) \tag{7}$$

We must now assign values to the parameters α_{tip} and V_{WF}.

In Supplementary Note 2, we discussed the central role of screening in the tip-induced potential experienced by an antidot located at the graphene device edge. We observed that both C_{tip} and C_{bg} changed as a function of y_{tip}, leading to the peculiar tip-potential evolution extracted in Supplementary Fig. 4a, at $V_{tip} = -3$ V. From this map, it appears that no value of V_{tip} can exactly fulfill the condition of Supplementary Eq. (4) since the effective tip-induced potential is different above graphene and outside the sample. We therefore had to estimate the work function value from the data of Supplementary Fig. 4. By took as criterion that the mean ΔV_{bg} along y_{tip} should be close to 0. It led us to take $V_{WF} \sim -3$ V.

Finally, we estimated α_{tip} from Fig. 3b of the manuscript. In this figure, the Lorentzian fit gave us $\Delta V_{bg} = -1.55$ V. Considering the work function value determined just above, we obtained from Supplementary Eq. (5) that $\alpha_{tip} \sim 2$.

From these numerical values (such as the value of α given in Supplementary Note 1), Supplementary Eq. (7) gives us a direct link between the tip-induced decrease of holes density and the tip bias expressed as

$$|\Delta n_{tip}| \sim (V_{tip} + 3) \times 4.7 \sim 10^{14}\ \mathrm{m}^{-2}\mathrm{V}^{-1}. \quad (8)$$

Supplementary Note 6: The backscattering regimes through an antidot

In this section, we discuss in more details the different regimes of coupling between up- and downstream QHECs through an antidot. Indeed, the longitudinal resistance R_{xx} reflects the backscattering of charge carriers tuned by the transmission T between up- and downsteam QHECs. This transmission can be expressed as

$$T = T_{da} T_a T_{au} \quad (9)$$

where T_{da} is the transmission between a downsteam QHEC and the antidot, T_a is the transmission through the antidot and T_{au} is the transmission between the antidot and the upstream QHEC.

We now discuss three regimes linked to the radius R of the antidot (see Supplementary Fig. 7). This radius is varied by changing either the Fermi energy E_F compared to the Landau levels (through a change of the back-gate voltage V_{bg}), either through the local change of potential induced by the carge tip, as discussed in Fig. 4 of the manuscript.

In the first regime (Supplementary Figs. 7a,e), the antidot is tunnel-coupled to the down- and upstream QHECs. The corresponding transmission coefficients are respectively $0 < T_{da} < 1$ and $0 < T_{au} < 1$. In this case, the total transmission T of Supplementary Eq. (9) is given by the transmission T_a in the antidot. As detailed in the manuscript, $T_a = 1$ when one of the discrete energy levels of the antidot is aligned with the Fermi level (Fig. 2c of the manuscript) and $T_a = 0$ otherwise (Fig. 2b of the manuscript). It results in a succession of peaks in R_{xx} as the antidot's radius R increases (Supplementary Fig. 7j). The maximal values of these peaks increases with R since the transmissions T_{da} and T_{au} increases when the QHEC loop associated to the antidot draws nearer form the down- and upstream QHECs.

In the second regime (Supplementary Figs. 7b,f), the antidot is so large that it merges with the upstream. Therefore $T_{au} = 1$. In this situation, the antidot is no more a closed system and does not carry discrete energies so that $T_a = 1$. The transmission T only varies with T_{da} that increases with R (Supplementary Fig. 7j).

I the third regime (Supplementary Figs. 7c,g), the antidot still enlarges and merges with both the up- and downstream QHECs. Therefore, $T_{da} = T_a = T_{au} = 1$. In that case, the backscattering between down- and upstream QHECs is direct. In the case of a perfect coupling between the upstream QHEC and the injection contact, R_{xx} is expected to vanish (Supplementary Fig. 7k, orange line). In this situation, the filling factor is given by the bulk and is therefore around $\nu = -2$. However, if the upstream QHEC is only partially coupled with the injection contact [1], R_{xx} remains finite (Supplementary Fig. 7k, blue line). This mechanism is a good candidate to explain the wide transition between two zero R_{xx} regions in the $R_{xx} - V_{bg}$ curve in Fig. 1b of the manuscript and Supplementary Fig. 3b. Finally, in the fourth regime (Supplementary Figs. 7d,h), the upstream QHEC disappears, such as the second downstream QHEC. R_{xx} then vanishes (Supplementary Fig. 7l) and the filling factor is around $\nu = -2$.

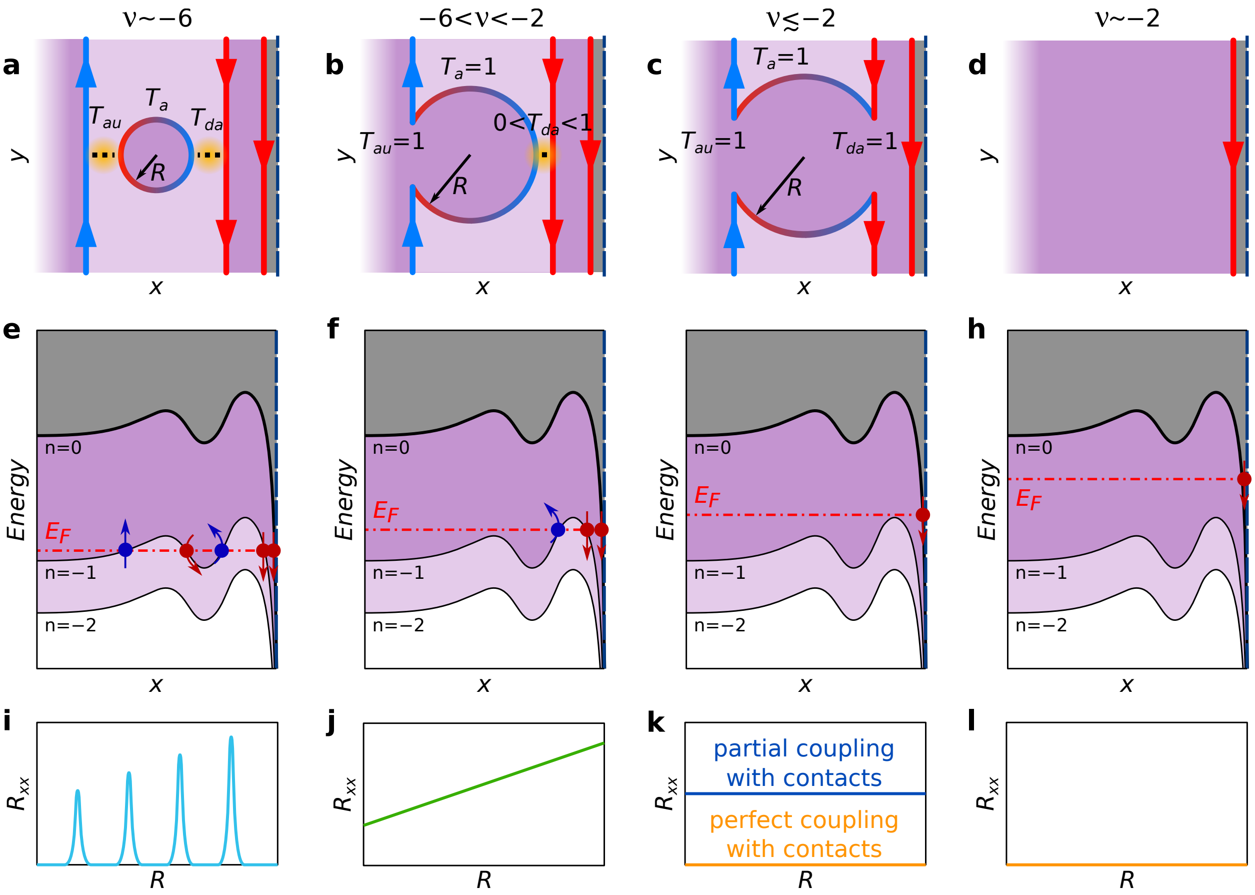

Supplementary Figure 7: Different scenario of transmission between the up- and downstream QHECs through an antidot. The downstream QHECs are represented in red and flow along the device edge (located downwards in each schematic). The upstream mode is in blue. The coupling between QHECs are represented with dotted lines and orange halos. Dark purple indicates an integer filling factor of -2 and light purple -6. **a**, The antidot is coupled to the upstream QHEC when the transmission $T_{au} > 0$ and to the nearest downstream QHEC when $T_{da} > 0$. The two downstream QHECs can be equilibrated when the transmission $T_{dd} > 0$. **b**, When the antidot is enlarged (by changing the potential in the vicinity of the antidot with the biased tip for instance), the QHEC loop of the antidot can be merged with the up- and downstream QHECs so that $T_{da} = T_{au} = 1$. If the two downstream QHEC are not equilibrated at all ($T_{dd} = 0$), then R_{xx} should indeed be zero. **c**, By considering that the potential is not symmetric on both sides of the antidot due to band bending (**f**), $T_{da} < T_{au}$. In this situation, while $T_{au} = 1$, the downstream QHEC is not fully equilibrated with the upstream QHEC and R_{xx} is not zero. **d-f**, Schematics of the three first Landau levels in the vicinity of the antidot. Due to band bending, the potential is not symmetric on both sides of the antidot.

Finally, in the fourth regime (Supplementary Figs. 7d,h), the upstream QHEC disappears, such as the second downstream QHEC. R_{xx} then vanishes (Supplementary Fig. 7l) and the filling factor is around $\nu = -2$.

Supplementary Note 7: Details on simulations

In this section, we detail the simulation methods. We first explain the choice of the tight-binding formalism to perform our simulation as well as the limitations inherent to this method. We then develop the tight-binding system used to reproduce the experimental results. We finish by presenting two different models that capture the main experimental features.

7.1 Tight-binding modelization and the absence of Coulomb energy

As precised in the main text, simulations were performed in the tight-binding framework using the KWANT package [17]. Whereas this package does not provide a recursive Poisson-Schrödinger solver, purely quantum mechanical-based calculations can capture the main physics we want to explore : coupling between QHECs via a localized state (an antidot). The major difference between simulations and the experiment therefore lays in the absence of Coulomb blockade in the antidot. Discrete energy levels originate only from the size confinement of the antidot (quantum terms E_n in Supplementary Eq. (2)). We therefore expect a qualitative correspondence between experiment and simulations, with the appearance of high R_{xx} peaks when one of the discrete energy level aligns with the Fermi energy but we also expect a significantly quantitative difference in the energy spacing between two peaks, since Coulomb energy is neglected in simulations. This is indeed observed in Fig. 4 of the manuscript.

7.2 Construction of the tight-binding model

In this section, we detail the tight-binding model we developed in order to simulate our experiment. Because the computation time increases fast with the number of atoms in the system [17], we can not simulate the whole sample. We therefore had to focus on a small region.

7.2.1 The tight-binding Hamiltonian

The tight-binding Hamiltonian can be expressed in the second quantization formalism as [18]

$$\hat{\mathcal{H}} = \sum_i U(\mathbf{r}_i)\hat{c}_i^\dagger \hat{c}_i - \sum_{<i,j>} t\, \hat{c}_i^\dagger \hat{c}_j \tag{10}$$

where $t = 2.7$ eV is the hopping parameter, $< i, j >$ is the restriction over the nearest neighboring atoms indices, $\hat{c}_i^\dagger$ and $\hat{c}_i$ are respectively the creation and annihilation operators on site i and $U(\mathbf{r}_i)$ is the potential on site i. For an hexagonal lattice as graphene, the energy dispersion at low energies is linear as a function of the wavevector $\mathbf{k}$ such that

$$E = \pm \frac{3at}{2}|\mathbf{k}| \tag{11}$$

where $a = 1.42$ Å is the inter-atomic distance of graphene. In this expression, the constant ratio between E and $|\mathbf{k}|$ is directly proportional to the Fermi velocity $v_F = 3at/2\hbar$. It is therefore possible to apply a scaling on a to reduce the number of atom in the studied system while keeping both the Fermi energy E_F and the Fermi wavelength k_F of charge carriers unchanged. It implies to define the following scaled quantities [18]

$$a_{\text{scaled}} = a \times s \qquad \text{and} \qquad t_{\text{scaled}} = t/s \tag{12}$$

where s is the scaling factor.

The magnetic field B is introduced in the tight-binding Hamiltonian of Supplementary Eq. (10) by modifying the hopping parameter t as

$$t \longrightarrow t \exp\left(-j\frac{e}{\hbar}B(x_i - x_j)\frac{y_i + y_j}{2}\right) \tag{13}$$

where (x_i, y_i) are the coordinates of site i. In graphene, the scaling transformations of Supplementary Eq. (12) also keeps LL energies unchanged while varying s at constant magnetic field B. Indeed, these energies are given by [19]

$$E_n = v_F\sqrt{2\hbar e}\sqrt{nB} \tag{14}$$

where $n = 0, \ \pm 1, \ \pm 2, ...$ is the LL label ($+$ for electrons and $-$ for holes).

Since k_F and E_n does not change with the scaling factor s, the transport simulation outputs will not be affected by the choice of s at a given E_F. Concerning the Fermi energy, we chose to change the onsite parameter $U(\mathbf{r}_i)$ in Supplementary Eq. (10) while keeping $E_F = 0$ to adjust the charge carrier density. For a uniform potential, this is equivalent to adjusting the charge carrier density by changing E_F with $U = 0$ eV. This is illustrated by Supplementary Fig. 8. To define hole-type charge carriers, the potential should be positive ($U > 0$).

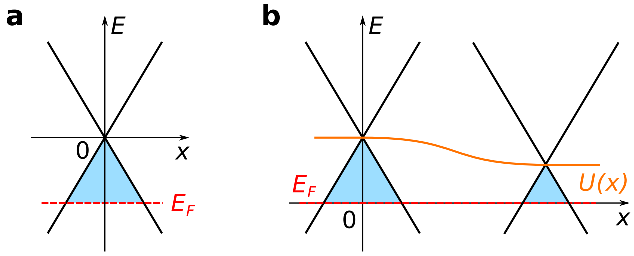

Supplementary Figure 8: The density and the type of charge carriers can be defined in two equivalent ways. a, Hole-type charge carriers are obtained by defining a negative E_F with the onsite parameter $U = 0$ eV. **b**, Hole-type charge carriers are obtained by defining a positive onsite parameter with a zero Fermi energy $E_F = 0$ eV. Compared to the situation depicted in **a**, it has the advantage to easily change the charge carrier density in the space.

The tip-induced potential is modeled with a Lorentzian function added to the onsite term of Supplementary Eq. (10). The function is given by

$$V_{tip}(\mathbf{r}_i) = \frac{V_{max}}{1 + \left(\frac{\mathbf{r_i} - \mathbf{r}_{tip}}{R_{tip}}\right)^2} \tag{15}$$

where $\mathbf{r}_i$ is the position of site i, V_{max} is the tip potential maximum, $\mathbf{r}_{tip}$ is the tip position in the $x - y$ plane and R_{tip} is half width at half maximum. From the experimental value (see Fig. 3b of the manuscript), we took $R_{tip} = 200$ nm (we didn't include the potential deformation due to screening). Here, V_{max} is directly related to the tip-induced change of charge carriers density Δn_{tip} discussed in Supplementary Note 5 for the experiment. To compare the simulations and the experiment, we need to find a correspondence between the charge density and the energy.

The relation between the carrier density $n(\mathbf{r}_i)$ and the onsite parameter $U(\mathbf{r}_i)$ is simply given by [18]

$$n(\mathbf{r}_i) = \frac{1}{\pi}\left(\frac{U(\mathbf{r}_i)}{\hbar v_F}\right)^2 \tag{16}$$

By expressing the Fermi velocity in terms of a and t ($v_F = 3at/2\hbar$), Δn_{tip} can easily be found by

$$\Delta n_{tip} = \frac{4}{9\pi}\left(\frac{U_{bulk} + V_{max}}{at}\right)^2 - \frac{4}{9\pi}\left(\frac{U_{bulk}}{at}\right)^2 \tag{17}$$

where U_{bulk} is the uniform onsite potential in the graphene bulk, as will be detailed in Supplementary Fig. 9d of the next section.

7.2.2 Geometrical definition of the simulated system

As discussed in detail in the manuscript, only the counterpropagating QHECs, located along the device edges, contribute to charge carriers transport, and the bulk is insulating. Furthermore, charge carriers flow without dissipation in forward propagating QHECs except when an antidot induces a coupling between forward and backward propagating channels (the coupling is determined by the transmission T of Supplementary Eq. (9). To capture the physics of the problem with simulations, we can therefore focus only on a small region along one of the device edges in which an antidot is located between the counterpropagating channels, as depicted in Supplementary Fig. 9a. A schematic view of the energy landscape with a high magnetic field applied perpendicularly to the studied region is presented in Supplementary Fig. 9b. Forward (red) and backward (blue) propagating QHECs appear at the intersection between the Fermi energy and the first Landau level (LL). An antidot (QH loop) is created due to a bump in the potential.

In order to reach the situation depicted in Supplementary Fig. 9b, we designed the potential represented in Supplementary Fig. 9c and Fig. 4e in the manuscript. This potential corresponds to the onsite term $U(\mathbf{r}_i)$ in Supplementary Eq. (10. The potential profile shown in Supplementary Fig. 9d (or Fig. 4f in the manuscript) highlights the presence of an antidot located between the counterpropagating QHECs. The QHECs emerge at the crossing between the LLs and the Fermi energy E_F. In particular, the forward QHEC (red arrow at the bottom of Supplementary Fig. 9c) appears along the device border due to edge confinement (hard wall potential). In the same way, a ”parasitic” backward channel (blue arrow at the top of Supplementary Fig. 9c) exists where the bulk is supposed to be insulating. We avoid the contribution from this QHEC by only computing transport from the left towards the right lead in the inset of Supplementary Fig. 9a. In this simulation, we ignore the coupling between QHECs and the ohmic contacts since we only concentrate on the coupling between up- and downstream QHEC through the antidot, determined by the transmission T of Supplementary Eq. (9).

Simulations of the whole sample, including the study of the coupling between QHECs and the ohmic contacts, are presented in [1]. Nevertheless, the large size of the simulated sample in this study does not allow to have a precise characterization of the transmission through the antidot, that is perfectly captured in the system of Supplementary Fig. 9.

7.2.3 The different studied systems

We simulated different systems to check that the observed results were resilient to changes of magnetic field, antidot size and modeling of the QHECs. Here, two systems of different size are

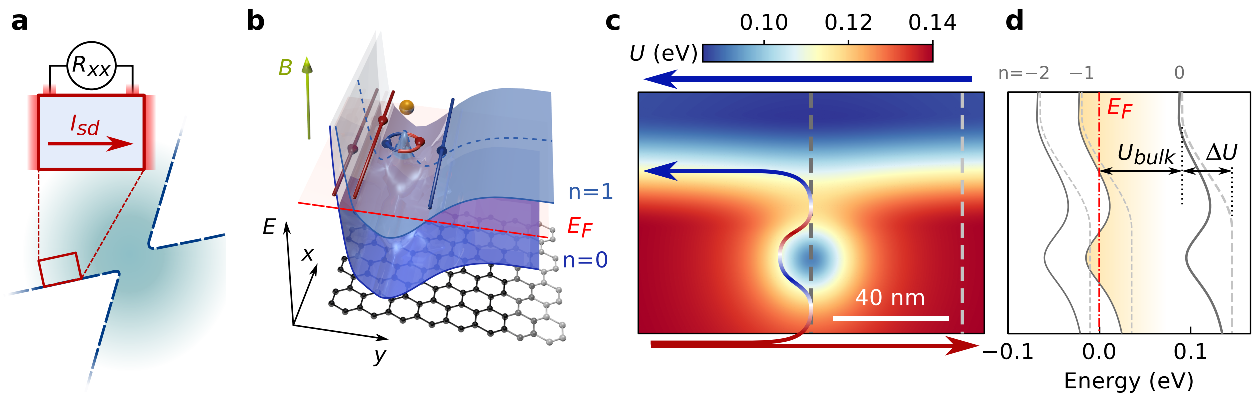

Supplementary Figure 9: Definition of the simulated system a, The simulated region is defined as the red rectangle. It is chosen to capture the physics along one of the edges of the whole sample used in the experiment. **b**, Artist's view of the physics we want to examine in the region defined in **a**. More details in Fig. 2a of the manuscript. **c**, Potential landscape (term $U(\mathbf{r}_i)$ in Supplementary Eq. (10), similar to Fig. 4e of the manuscript. The arrows illustrate the charge carriers flow in QHECs (forward in red and backward in blue). QHECs appear at the natural graphene edges (bottom red and top blue arrows). By considering only left to right transport, the backward top channel does not contribute. **d**, Three lowest Landau levels (LLs) along the dotted lines of **c**. The dark gray curves represent the LLs passing by the antidot and the light gray dashed curves represent the LLs that are not affect by the antidot potential. U_{bulk} gives the potential value in the bulk and $\Delta U = \zeta U_{bulk}$ models the increase of charge density at the edges. Here, we chose arbitrarily $\zeta = 0.6$. The profiles in the potential U of Fig. **c** are depicted with bold lines (coinciding with the $n = 0$ LL).

detailed, including the system presented in the manuscript.

The dimensions of the first studied system are 150×250 nm^2 and the antidot is modeled with a Gaussian function centered 45 nm away from the edge and having a FWHM of 72 nm (see Fig. 4d of the manuscript). A scaling factor $s = 4$ (Supplementary Eq. (12) has been used to decrease the computation time. The R_{xx} curve as a function of U_{bulk} is shown in Supplementary Fig. 10. The R_{xx} curve as a function of Δn_{tip} presented in Supplementary Fig. 10b (same as Fig. 4c of the manuscript) is obtained for $U_{bulk} = 0.0895$ eV (red arrow in Supplementary Fig. 10a).

The second simulated system is presented in Supplementary Figs. 9c,d. The dimensions are 85×120 nm^2 and the antidot is modeled by a Gaussian function of full width at half maximum (FWHM) of 28 nm and centered 26.5 nm from the edge. No scaling was applied to the inter-atomic distance ($a = 1.42$ Å) and to the hopping parameter ($t = 2.7$ eV). R_{xx} as a function of Δn_{tip} obtained with a magnetic field of 12 T are presented in Supplementary Fig. 11a. As for the first situation, R_{xx} peaks appear by increasing the tip perturbation. However, the spacing between the peaks is wider than for the larger antidots (Supplementary Fig. 10b). This is expected since the quantum discrete energy levels move apart from each others when decreasing the size of the localized state.

The local current density for different values of Δn_{tip} are presented in Supplementary Figs. 11b-e. As discussed in the manuscript, the current density in the antidot is higher on a R_{xx} peak (Supplementary Fig. 11c) than at zero R_{xx} (Supplementary Fig. 11b). It highlights that R_{xx} peaks coincide with resonance conditions in the antidot. As detailed in Fig. 4i of the manuscript, the finite R_{xx} plateau correspond to direct backscattering between the forward and the backward QHEC.

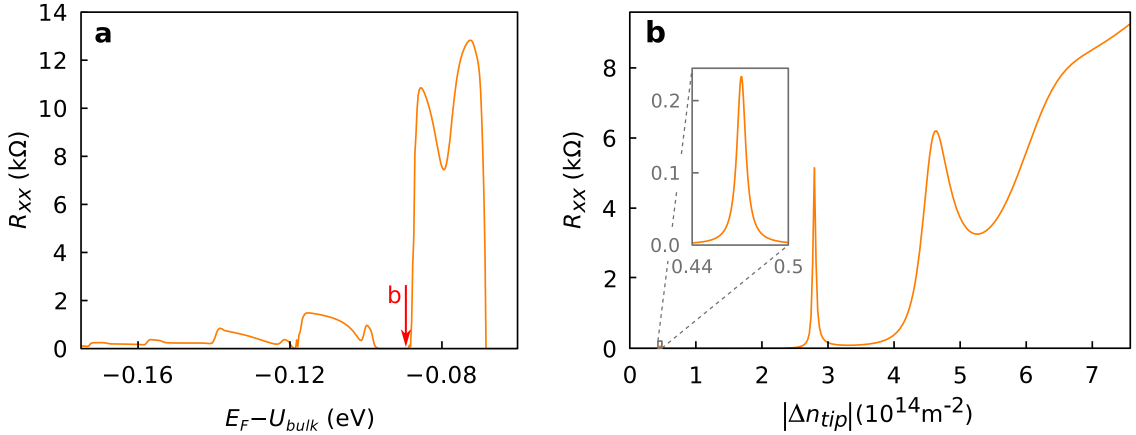

Supplementary Figure 10: Transport measurements in the first studied system. **a**, R_{xx} as a function of U_{bulk} (see Supplementary Fig. 9d). **b**, R_{xx} as a function of the maximal tip-induced change of hole density $|\Delta n_{tip}|$ with $U_{bulk} = 0.0895$ eV (red arrow in **a**).

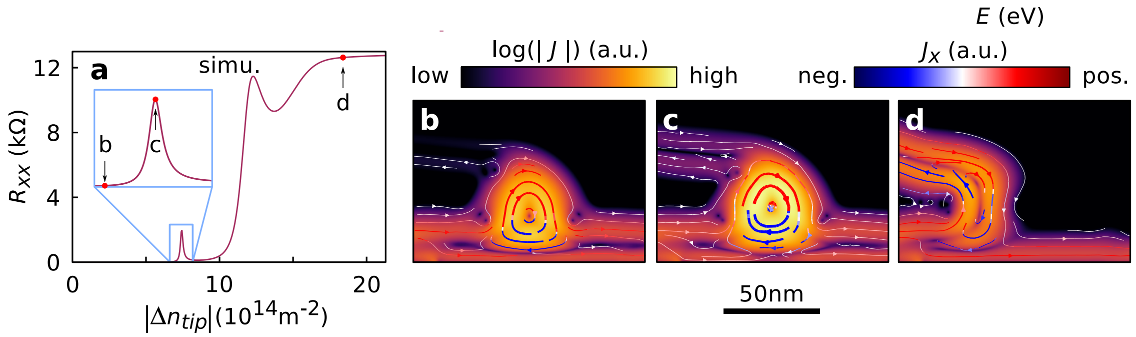

Supplementary Figure 11: Transport measurements in the second studied system. **a**, R_{xx} as a function of the maximal tip-induced change of hole density $|\Delta n_{tip}|$ with $U_{bulk} = 0.089$ eV obtained with the potential presented in Supplementary Figs. 9c,d. **b-d**, Local current density maps obtained for the values Δn_{tip} indicated with arrows in **a**.

References

[1] Moreau, N. *et al.* Contacts and upstream modes explain the electron-hole asymmetry in the graphene quantum hall regime (2021). 2103.10331.

[2] Cui, Y.-T. *et al.* Unconventional correlation between quantum hall transport quantization and bulk state filling in gated graphene devices. *Phys. Rev. Lett.* **117**, 186601 (2016). URL https://link.aps.org/doi/10.1103/PhysRevLett.117.186601.

[3] Schnez, S., Güttinger, J., Stampfer, C., Ensslin, K. & Ihn, T. The relevance of electrostatics for scanning-gate microscopy. *New J. Phys.* **13**, 053013 (2011). URL https://iopscience.iop.org/article/10.1088/1367-2630/13/5/053013.

[4] Gutiérrez, C. *et al.* Interaction-driven quantum hall wedding cake–like structures in graphene quantum dots. *Science* **361**, 789–794 (2018). URL https://science.sciencemag.org/content/361/6404/789.

[5] Walkup, D. *et al.* Tuning single-electron charging and interactions between compressible landau level islands in graphene. *Phys. Rev. B* **101**, 035428 (2020). URL https://link.aps.org/doi/10.1103/PhysRevB.101.035428.

[6] Hanson, R., Kouwenhoven, L. P., Petta, J. R., Tarucha, S. & Vandersypen, L. M. K. Spins in few-electron quantum dots. *Rev. Mod. Phys.* **79**, 1217–1265 (2007). URL https://link.aps.org/doi/10.1103/RevModPhys.79.1217.

[7] Rosenow, B. & Halperin, B. I. Influence of interactions on flux and back-gate period of quantum hall interferometers. *Phys. Rev. Lett.* **98**, 106801 (2007). URL https://link.aps.org/doi/10.1103/PhysRevLett.98.106801.

[8] Ihnatsenka, S., Zozoulenko, I. V. & Kirczenow, G. Electron-electron interactions in antidot-based aharonov-bohm interferometers. *Phys. Rev. B* **80**, 115303 (2009). URL https://link.aps.org/doi/10.1103/PhysRevB.80.115303.

[9] Ofek, N. *et al.* Role of interactions in an electronic fabry–perot interferometer operating in the quantum hall effect regime. *Proceedings of the National Academy of Sciences* **107**, 5276–5281 (2010). URL https://www.pnas.org/content/107/12/5276. https://www.pnas.org/content/107/12/5276.full.pdf.

[10] Yu, Y.-J. *et al.* Tuning the graphene work function by electric field effect. *Nano Lett.* **9**, 3430–3434 (2009). URL https://doi.org/10.1021/nl901572a.

[11] Silvestrov, P. G. & Efetov, K. B. Charge accumulation at the boundaries of a graphene strip induced by a gate voltage: Electrostatic approach. *Phys. Rev. B* **77**, 155436 (2008). URL https://link.aps.org/doi/10.1103/PhysRevB.77.155436.

[12] Vera-Marun, I. J. *et al.* Quantum hall transport as a probe of capacitance profile at graphene edges. *Applied Physics Letters* **102**, 013106 (2013). URL https://doi.org/10.1063/1.4773589. https://doi.org/10.1063/1.4773589.

[13] Barraud, C. *et al.* Field effect in the quantum hall regime of a high mobility graphene wire. *Journal of Applied Physics* **116**, 073705 (2014). URL https://doi.org/10.1063/1.4893468. https://doi.org/10.1063/1.4893468.

[14] Lai, K. *et al.* Imaging of coulomb-driven quantum hall edge states. *Phys. Rev. Lett.* **107**, 176809 (2011). URL https://link.aps.org/doi/10.1103/PhysRevLett.107.176809.

[15] Marguerite, A. *et al.* Imaging work and dissipation in the quantum hall state in graphene. *Nature* **575**, 628–633 (2019). URL https://doi.org/10.1038/s41586-019-1704-3.

[16] Brun, B. *et al.* Imaging dirac fermions flow through a circular veselago lens. *Phys. Rev. B* **100**, 041401 (2019). URL https://link.aps.org/doi/10.1103/PhysRevB.100.041401.

[17] Groth, C. W., Wimmer, M., Akhmerov, R. A. & Waintal, X. Kwant: a software package for quantum transport. *New J. Phys.* **26**, 063065 (2014). URL https://iopscience.iop.org/article/10.1088/1367-2630/16/6/063065.

[18] Liu, M.-H. *et al.* Scalable tight-binding model for graphene. *Phys. Rev. Lett.* **114**, 036601 (2015). URL https://link.aps.org/doi/10.1103/PhysRevLett.114.036601.

[19] Goerbig, M. O. Electronic properties of graphene in a strong magnetic field. *Rev. Mod. Phys.* **83**, 1193–1243 (2011). URL https://link.aps.org/doi/10.1103/RevModPhys.83.1193.
